# Comparison of the effects of forefoot joint-preserving arthroplasty and resection-replacement arthroplasty on walking plantar pressure distribution and patient-based outcomes in patients with rheumatoid arthritis

**DOI:** 10.1371/journal.pone.0183805

**Published:** 2017-08-29

**Authors:** Kosuke Ebina, Makoto Hirao, Keishi Takagi, Sachi Ueno, Tokimitsu Morimoto, Hozo Matsuoka, Kazuma Kitaguchi, Toru Iwahashi, Jun Hashimoto, Hideki Yoshikawa

**Affiliations:** 1 Department of Orthopaedic Surgery, Osaka University Graduate School of Medicine, Suita, Osaka, Japan; 2 Department of Rehabilitation, Osaka University Graduate School of Medicine, Suita, Osaka, Japan; 3 Department of Orthopaedic Surgery, Suita Municipal Hospital, Suita, Osaka, Japan; 4 Department of Rheumatology, National Hospital Organization, Osaka-Minami Medical Center, Kawachinagano, Osaka, Japan; Georgia Regents University, UNITED STATES

## Abstract

**Purpose:**

The purpose of this retrospective study is to clarify the difference in plantar pressure distribution during walking and related patient-based outcomes between forefoot joint-preserving arthroplasty and resection-replacement arthroplasty in patients with rheumatoid arthritis (RA).

**Methods:**

Four groups of patients were recruited. Group1 included 22 feet of 11 healthy controls (age 48.6 years), Group2 included 36 feet of 28 RA patients with deformed non-operated feet (age 64.8 years, Disease activity score assessing 28 joints with CRP [DAS28-CRP] 2.3), Group3 included 27 feet of 20 RA patients with metatarsal head resection-replacement arthroplasty (age 60.7 years, post-operative duration 5.6 years, DAS28-CRP 2.4), and Group4 included 34 feet of 29 RA patients with metatarsophalangeal (MTP) joint-preserving arthroplasty (age 64.6 years, post-operative duration 3.2 years, DAS28-CRP 2.3). Patients were cross-sectionally examined by F-SCAN II to evaluate walking plantar pressure, and the self-administered foot evaluation questionnaire (SAFE-Q). Twenty joint-preserving arthroplasty feet were longitudinally examined at both pre- and post-operation.

**Results:**

In the 1^st^ MTP joint, Group4 showed higher pressure distribution (13.7%) than Group2 (8.0%) and Group3 (6.7%) (P<0.001). In the 2^nd^-3^rd^ MTP joint, Group4 showed lower pressure distribution (9.0%) than Group2 (14.5%) (P<0.001) and Group3 (11.5%) (P<0.05). On longitudinal analysis, Group4 showed increased 1^st^ MTP joint pressure (8.5% vs. 14.7%; P<0.001) and decreased 2^nd^-3^rd^ MTP joint pressure (15.2% vs. 10.7%; P<0.01) distribution. In the SAFE-Q subscale scores, Group4 showed higher scores than Group3 in pain and pain-related scores (84.1 vs. 71.7; P<0.01) and in shoe-related scores (62.5 vs. 43.1; P<0.01).

**Conclusions:**

Joint-preserving arthroplasty resulted in higher 1^st^ MTP joint and lower 2^nd^-3^rd^ MTP joint pressures than resection-replacement arthroplasty, which were associated with better patient-based outcomes.

## Introduction

Rheumatoid arthritis (RA) is frequently associated with painful foot deformities, which is reported in 75% of patients within four years of diagnosis, increasing to approximately 90% during the course of the disease [1, 2]. These deformities includes hallux valgus (HV), dorsal dislocation of the metatarsophalangeal (MTP) joints, and hammer toe deformity of the lesser toes [3–5], which are associated with disability in daily activities [6, 7] and considerable changes in plantar pressure intensity and its distribution pattern [8, 9]. Previous reports demonstrated that forefoot joint damage is associated with both high forefoot pressure and plantar pain during walking [9–12], and Vidmar et al. reported the reliability of in-shoe plantar pressure measurements during walking by the F-SCAN system (Tekscan Inc., Boston, MA) in RA patients [12].

There is a trend toward joint-preserving arthroplasty instead of conventional resection-replacement arthroplasty of forefoot deformities with recent advances in the pharmacological treatment of RA [13, 14]. Moreover, evaluation of clinical outcomes by a patient-based outcome instrument is recently becoming common in various orthopedic diseases and surgeries [15, 16], and the Japanese Society for Surgery of the Foot (JSSF) has recently developed a patient-based self-administered foot evaluation questionnaire (SAFE-Q) [17]. A previous report demonstrated that, compared to other foot diseases, patients with RA had the lowest subscale scores on the SAFE-Q, and the pain and pain-related subscale was more responsive than the SF-36 bodily pain subscale [17]. We have recently reported that forefoot joint-preserving arthroplasty resulted in better outcomes compared to resection-replacement arthroplasty on both SAFE-Q and radiographic assessments [18]. However, there have been no reports demonstrating the effects of forefoot surgery on the change of plantar pressure distribution and its relevance to patient-based outcomes.

The purpose of this retrospective study was to evaluate and compare the effects of both forefoot resection-replacement arthroplasty and joint-preserving arthroplasty on walking plantar pressure distribution and its relevance to patient-based outcomes in RA.

## Materials and methods

### Patients

Four groups of patients were recruited from April 2012 to December 2015. The recruiting criteria was all of the RA patients who underwent forefoot arthroplasty during this period, and RA patients without operation who had forefoot symptoms. Group1 included 22 feet of 11 healthy controls (age 48.6 years), Group2 included 36 feet of 28 RA patients with symptomatic deformed non-operated feet [age 64.8 years, Disease activity score assessing 28 joints with CRP (DAS28-CRP) 2.3], Group3 included 27 feet of 20 RA patients with metatarsal head resection-replacement arthroplasty (postoperative age 60.7 years, post-operative duration 5.6 years, DAS28-CRP 2.4, mainly Swanson implant replacement of the hallux MTP joint and metatarsal head resection of the lesser toes), and Group4 included 34 feet of 29 RA patients with metatarsophalangeal joint-preserving arthroplasty (postoperative age 64.6 years, post-operative duration 3.2 years, DAS28-CRP 2.3, mainly modified Scarf osteotomy of the hallux and off-set shortening osteotomy of the lesser toes) were enrolled. The operation procedures were selected at the discretion of each senior rheumatoid surgeon and performed from January 2000 to December 2015. In operated patients, postoperative evaluation was performed only for those who completed more than 6 months of follow-up.

### Assessment

**Fig 1:**
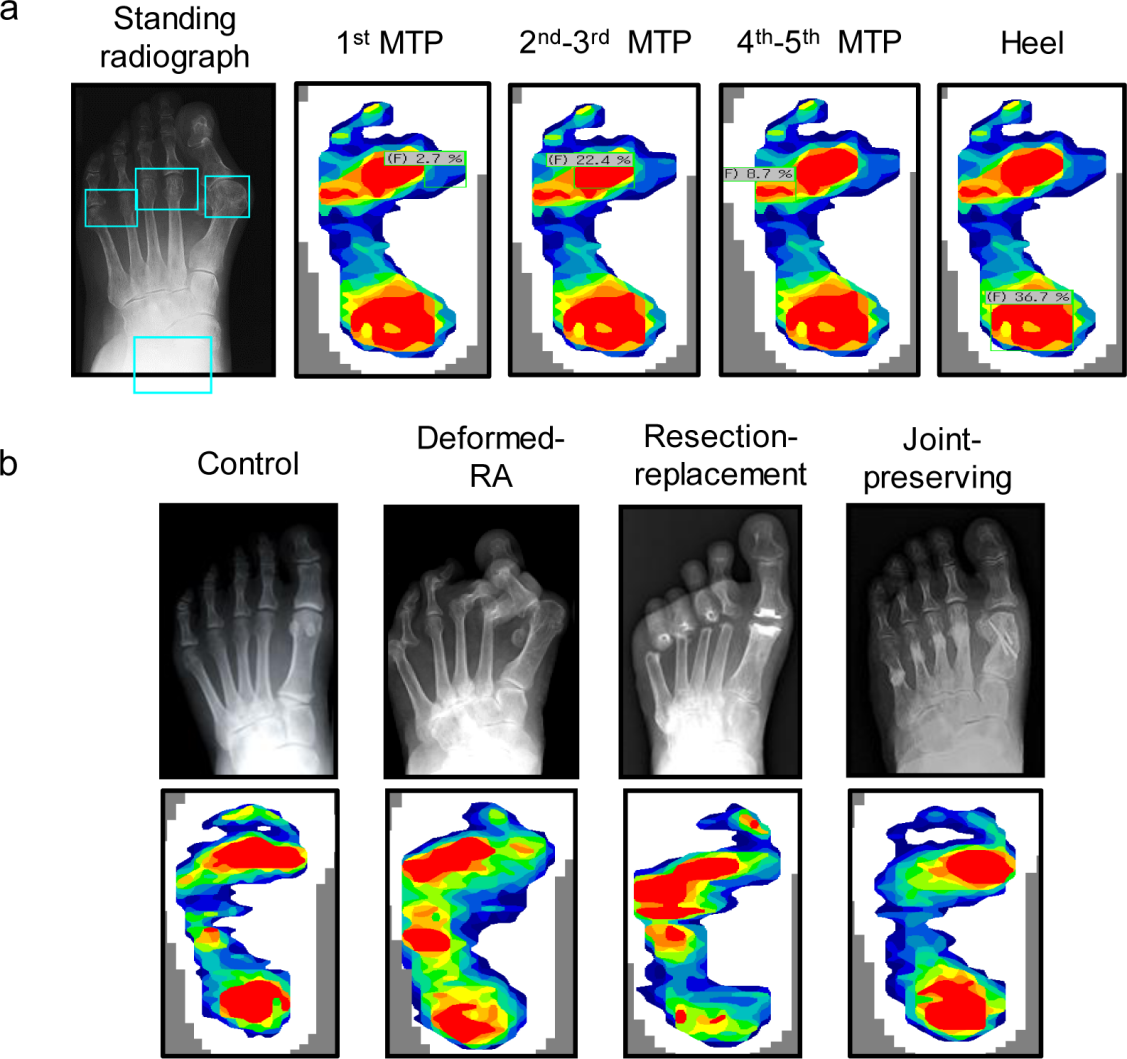
Plantar peak pressure distribution (%) of the region of interest matched with patients’ standing feet X-rays, and representative radiographs and plantar peak pressure distribution of each group. (a) Walking peak plantar pressure distribution of the 1^st^ MTP joint, 2^nd^-3^rd^ MTP joint, 4^th^-5^th^ MTP joint and the heel evaluated by the F-SCAN II system. (b) The healthy control, deformed-RA, metatarsal head resection-replacement arthroplasty (Swanson implant replacement of the hallux metatarsophalangeal joint and metatarsal head resection of the lesser toes), and metatarsophalangeal joint-preserving arthroplasty (modified Scarf osteotomy with inter-positioning technique of the medical capsule of the hallux and off-set shortening osteotomy of the lesser toes) groups were evaluated.

Walking plantar pressures were evaluated using the F-SCAN II system (Nitta Co. Ltd., Tokyo, Japan) as previously described [19]. Briefly, an F-SCAN II insole (0.15-mm thick) with 960 force-sensing resistors (25 mm^2^ cells), which has relatively high resolution compared to other foot pressure measurement systems [20]. All subjects were given a warm-up period to acclimatize to the footwear, plantar pressure measurement was then performed in more than 8 consecutive walks without shoe orthoses, and the average data of 3–6 walks were used. The system software was used to generate gross peak pressure patterns [9], and regions of interest (ROIs) (1^st^ MTP joint, 2^nd^-3^rd^ MTP joint, 4^th^-5^th^ MTP joint, and heel) were defined by matching with each patients’ standing feet X-rays as shown in Fig 1A. The peak pressure distribution (%) of the ROI compared to the whole-foot peak pressure was evaluated as previously described [19].

At the same time as the F-SCAN II assessment, patients were radiographically evaluated and asked to answer the postoperative SAFE-Q. The main body of the outcome instrument consists of 34 questionnaire items, which provide five subscale scores (1: Pain and pain-related; 2: Physical functioning and daily living; 3: Social functioning; 4: Shoe-related; and 5: General health and well-being), and each subscale score ranges from 0 to 100 points [17].

The clinical characteristics of each group are shown in Table 1. The leg-heel angle, hallux valgus (HV) angle, first metatarsal and second metatarsal (M1M2) angle, and first metatarsal and fifth metatarsal (M1M5) angle were defined by weight-bearing radiographs. Among the operated RA patients, 5 feet of the resection-replacement group and 20 feet of the joint-preserving group were longitudinally evaluated at both before and after surgery with more than 6 months intervals, to investigate the effects of these operations on the change in plantar pressure distribution.

**Table 1:** Clinical characteristics and radiographic evaluation of each group.

Variable		Control (n = 22)	Deformed-RA (n = 36)	Resection-replacement (n = 27)		Joint-preserving (n = 34)	
Operation methods (n)	Hallux	-		Swanson implant (n = 26)		Modified Scarf (n = 29)	
				Metatarsal head resection (n = 1)		Modified Mann (n = 4)	
						Lapidus (n = 1)	
	Lesser toes	-		Metatarsal head resection (n = 27)		Off-set osteotomy (n = 34)	
Pre-op / Post-op (at evaluation)		-	-	Pre-op	Post-op (at evaluation)	Pre-op	Post-op (at evaluation)
Post-op duration (years)		-	-	-	5.6±0.8	-	3.2±0.5*
Age, (years)		48.6±3.8	64.8±2.0	54.8±1.3	60.7±1.5	61.2±1.6	64.6±1.7
Gender, Females (%; n/N)		54.5 (12/22)	91.7 (33/36)	100 (27/27)		88.2 (30/34)	
Body mass index (kg/m^2^)		21.6±0.6	21.0±0.6	20.7±0.7	20.3±0.6	21.7±0.6	21.9±0.6
Duration of disease (years)		-	20.7±2.0	20.3±1.4	26.0±1.5^#^	19.0±2.1	22.4±2.0
Steinbrocker’ s stage (n)		-	Ⅲ(n = 4) Ⅳ(n = 32)	Ⅲ(n = 1) Ⅳ(n = 26)		Ⅲ(n = 5) Ⅳ(n = 29)	
Steinbrocker’ s functional class (n)		-	Ⅱ(n = 12) Ⅲ(n = 24)	Ⅱ(n = 12) Ⅲ(n = 14) Ⅳ(n = 1)		Ⅱ(n = 18) Ⅲ(n = 16)	
RF positivity (%)		-	86.1 (31/36)	85.2 (23/27)	88.9 (24/27)	82.4 (28/34)	79.4 (27/34)
DAS28-CRP		-	2.3±0.1	2.6±0.1	2.4±0.2	2.4 ± 0.1	2 .3±0.1
Prednisolone dose (mg/day)		-	0.7±0.2	2.8±0.5^#^	3.0±0.8^#^	1.0±0.4*	0.6±0.2**
Prednisolone usage (%)		-	22.2 (8/36)	51.9 (14/27) ^#^	55.6 (15/27) ^#^	29.4 (10/34)*	23.5 (8/34)*
MTX dose (mg/week)		-	3.8±0.6	3.6±0.8	3.9±0.7	4.4±0.6	4.6±0.7
MTX usage (%)		-	63.9 (23/36)	51.9 (14/27)	55.6 (15/27)	73.5 (25/34)	70.6 (24/34)
Biologics usage (%)		-	47.2 (17/36)	18.5 (5/27) ^#^	22.2 (6/27) ^#^	38.2 (13/34)	41.2 (14/34)
Biologics (n)		-	TCZ(9) ETN(4) ABT(4)	TCZ(2) ETN(2) IFX(1)	TCZ(3) ETN(2) IFX(1)	TCZ(8) ETN(2) ABT(3)	TCZ(9) ETN(2) ABT(3)
Prior lower limb operation (n)		-	THA (n = 3) TKA (n = 9)	THA (n = 2) TKA (n = 6)	THA (n = 3) TKA (n = 8)	THA (n = 2) TKA (n = 4)	THA (n = 2) TKA (n = 5)
			TAA (n = 1)	Knee synovectomy (n = 2)	Knee synovectomy (n = 2)	TAA (n = 2)	TAA (n = 3)
			Subtalar arthrodesis (n = 1)				Subtalar arthrodesis (n = 1)
Leg-heel angle (degree)		-	3.0±1.1	-	2.7±1.7	-	2.5±1.1
HV angle (degree)		-	39.2±3.7^†††^	37.6 ± 3.3	19.0±2.0^###^	40.2 ± 2.8	11.5±1.8**
M1M2 angle (degree)		-	13.0±0.7^†††^	11.4 ± 1.0	7.6±0.9^###^	13.6 ± 0.7	8.2±0.7
M1M5 angle (degree)		-	33.2±1.4^†††^	34.6 ± 1.3	30.7±1.3	35.7 ± 1.0	23.1±0.9***

Mean ± Standard Error (SE), unless otherwise noted. N.S., not significant.

Pre-op, Pre-operation; Post-op, Post-operation; RF, Rheumatoid factor; DAS28-CRP, Disease activity score assessing 28 joints with CRP; MTX, Methotrexate; TCZ, tocilizumab; ETN, etanercept

ABT, abatacept; IFX, infliximab; THA, Total hip arthroplasty; TKA, Total knee arthroplasty; TAA, Total ankle arthroplasty; HV, Hallux valgus; M1M2, first metatarsal and second metatarsal; M1M5, first metatarsal and fifth metatarsal.

Differences between the groups were determined by ANOVA, the Mann-Whitney U-test, or the chi-squared test.

* P<0.05

** P<0.01

*** P<0.001; Resection-replacement vs Joint-preserving group

# P<0.05

### P<0.001; Deformed-RA vs Resection-replacement group

††† P<0.001; Deformed-RA vs Joint-preserving group

This study was conducted at single center and in accordance with the ethical standards of the Declaration of Helsinki which was approved by the Institutional Ethics Review Board (approval number: 14219; Osaka University, Graduate School of Medicine). Written, informed consent was obtained from each patient.

### Surgical procedure

Representative radiographs and plantar peak pressure distributions of each groups are shown in Fig 1B. As for the resection-replacement arthroplasty, most patients (96.3%; n = 26/27) were treated by the combination of Swanson implant replacement of the hallux with the medial approach [21] and metatarsal head resection osteotomy of the lesser toes with a dorsal or plantar approach, as previously described [22]. The medial capsule of the hallux was prepared as a rectangular-shaped flap and sutured onto the first metatarsal bone [21], and adductor hallucis was released from the great toe from the intra-articular side.

As for the joint-preserving arthroplasty, most patients (85.3%; n = 29/34) were treated by the combination of modified Scarf osteotomy of the hallux with the medial longitudinal approach [23] and off-set shortening osteotomy of the lesser toes with a dorsal longitudinal approach between the second and third toe MTP joint, and between the fourth and fifth toe MTP joint, as previously described [24]. The hallux was internally fixed with AcuTwist Acutrak 2.0-mm headless compression screws (Acumed USA, Hillsboro, OR) or 2.0–3.0-mm cannulated cortical screws. The medial capsule of the hallux was prepared as a rectangular-shaped flap and sutured to adductor hallucis with inter-positioning technique [25], which was released from the hallux from the extra-articular side.

In both groups, proximal interphalangeal (PIP) joint resection arthroplasty of the lesser toes with a dorsal approach was added if patients had rigid flexion deformities of the PIP joint, and the lesser toes were temporarily fixed with 1–1.2-mm-diameter Kirschner wires for 2–3 weeks [18]. After removal of the Kirschner wires, the patients were allowed to walk with arch support orthoses, and range of motion exercises were encouraged.

### Statistical analysis

Differences between the groups were tested using analysis of variance (ANOVA), the Mann-Whitney *U* test, or the chi-squared test, as appropriate. Changes in each score from before to after surgery at specified time points within each study group were compared using the nonparametric Wilcoxon signed-rank test. Results are expressed as means ± standard error. A P value < 0.05 indicated significance. All tests were performed using IBM SPSS Statistics version 22 software (IBM, Armonk, NY).

## Results

Patients’ clinical characteristics and operation-related outcomes of each group when performing F-SCAN II are shown in Table 1. Generally, patients in the control group were younger and included more males compared to RA groups. In addition, patients with higher prednisolone dose (3.0 vs. 0.6 mg/day; P<0.01) and higher prednisolone usage (55.6 vs. 23.5%; P<0.05) tended to be treated with resection-replacement arthroplasty rather than joint-preserving arthroplasty. No significant differences were observed in age, duration of disease, DAS28-CRP, methotrexate (MTX) dose and usage, and biologic usage between the resection-replacement group and the joint-preserving group.

Representative X-ray and plantar peak pressure distributions are shown in Fig 1B. The control and joint-preserving groups tended to show medial loading and a high 1st MTP joint pressure, although the deformed-RA and resection-replacement groups tended to show lateral loading and a small 1^st^ MTP joint pressure. As for radiographic parameters, the HV angle (19.0° vs. 11.5°; P<0.01) and the M1M5 angle (30.7° vs. 23.1°; P<0.001) were significantly smaller in the joint-preserving group than in the resection-replacement group (Table 1).

**Fig 2:**
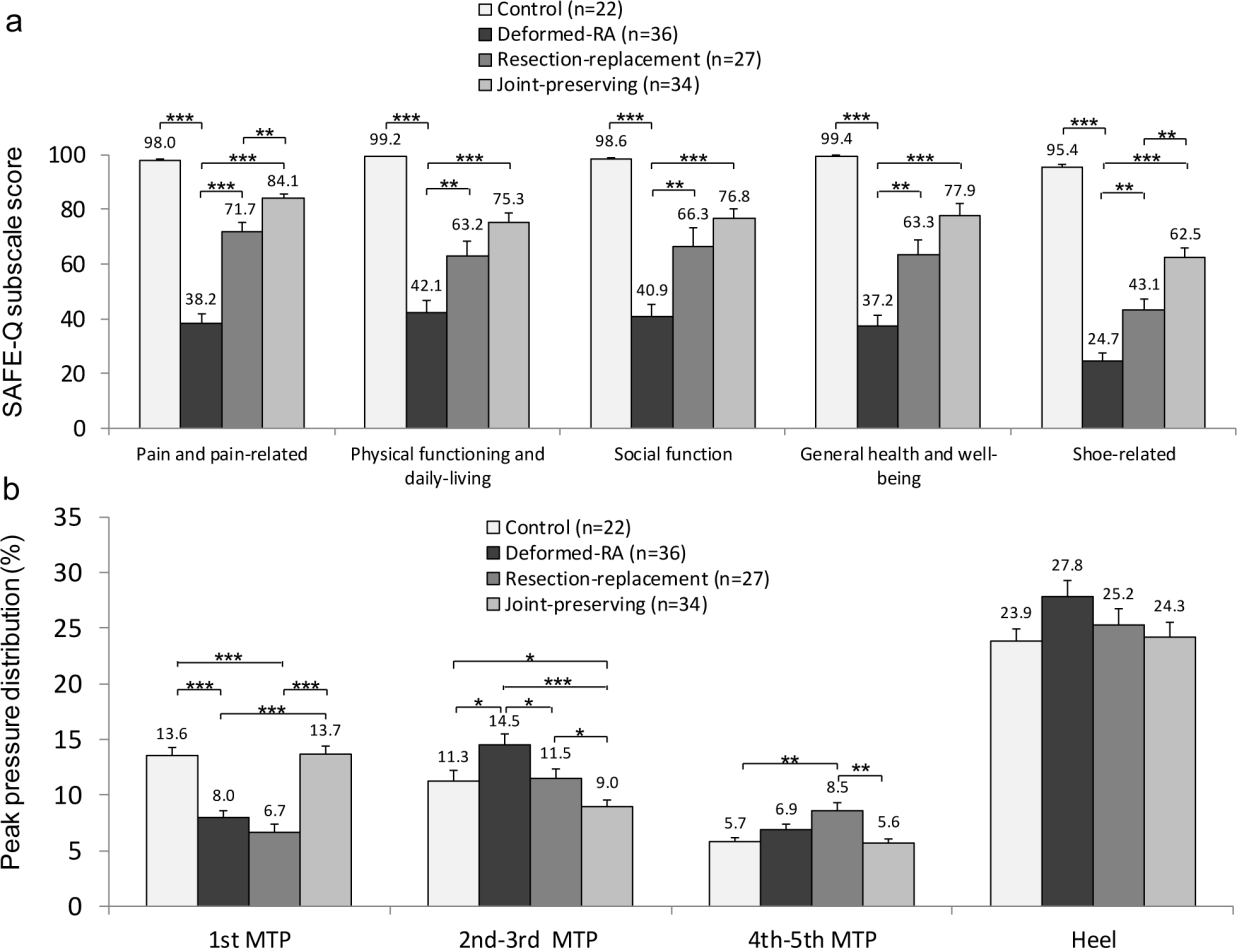
Mean SAFE-Q subscale scores and plantar peak pressure distribution (%) of each group. (a) Pain and pain-related scores, physical functioning and daily-living scores, social functioning scores, general health and well-being scores, and shoe-related scores were evaluated (full score 100 points). (b) 1^st^ MTP joint, 2^nd^-3^rd^ MTP joint, 4^th^-5^th^ MTP joint, and heel distributions were evaluated. Bars indicate standard errors. * P < 0.05, ** P < 0.01, and *** P < 0.001 between the groups.

Mean SAFE-Q subscale scores (full score 100 points) are shown in Fig 2A. Compared to the control group, all subscale scores were significantly lower in the deformed-RA group (P<0.001), and both the resection-replacement group and the joint-preserving group showed significantly higher subscale scores compared to the deformed-RA group (P<0.01-P<0.001). However, the joint-preserving group showed significantly higher scores compared to the resection-replacement group in pain and pain-related scores (84.1 vs. 71.7 points; P<0.01) and in shoe-related scores (62.5 vs. 43.1 points; P<0.01).

Peak pressure distributions (%) of the ROIs (1^st^ MTP joint, 2^nd^-3^rd^ MTP joint, 4^th^-5^th^ MTP joint, and heel) are shown in Fig 2B. Compared to the control group, the deformed-RA group showed lower 1^st^ MTP joint pressure (13.6% vs. 8.0%; P<0.001) and higher 2^nd^-3^rd^ MTP joint pressure (11.3% vs. 14.5%; P<0.05). Compared to the deformed-RA group, the resection-replacement group showed similar 1^st^ MTP joint pressure (8.0% vs. 6.7%), but lower 2^nd^-3^rd^ MTP joint pressure (14.5% vs. 11.5%; P<0.05). Finally, compared to the resection-replacement group, the joint-preserving group showed higher 1^st^ MTP joint pressure (6.7% vs. 13.7%; P<0.001) and lower 2^nd^-3^rd^ MTP joint (11.5% vs. 9.0%; P<0.05) and 4^th^-5^th^ MTP joint (8.5% vs. 5.6%; P<0.05) pressures. No significant differences were observed in heel pressure between the groups.

**Fig 3:**
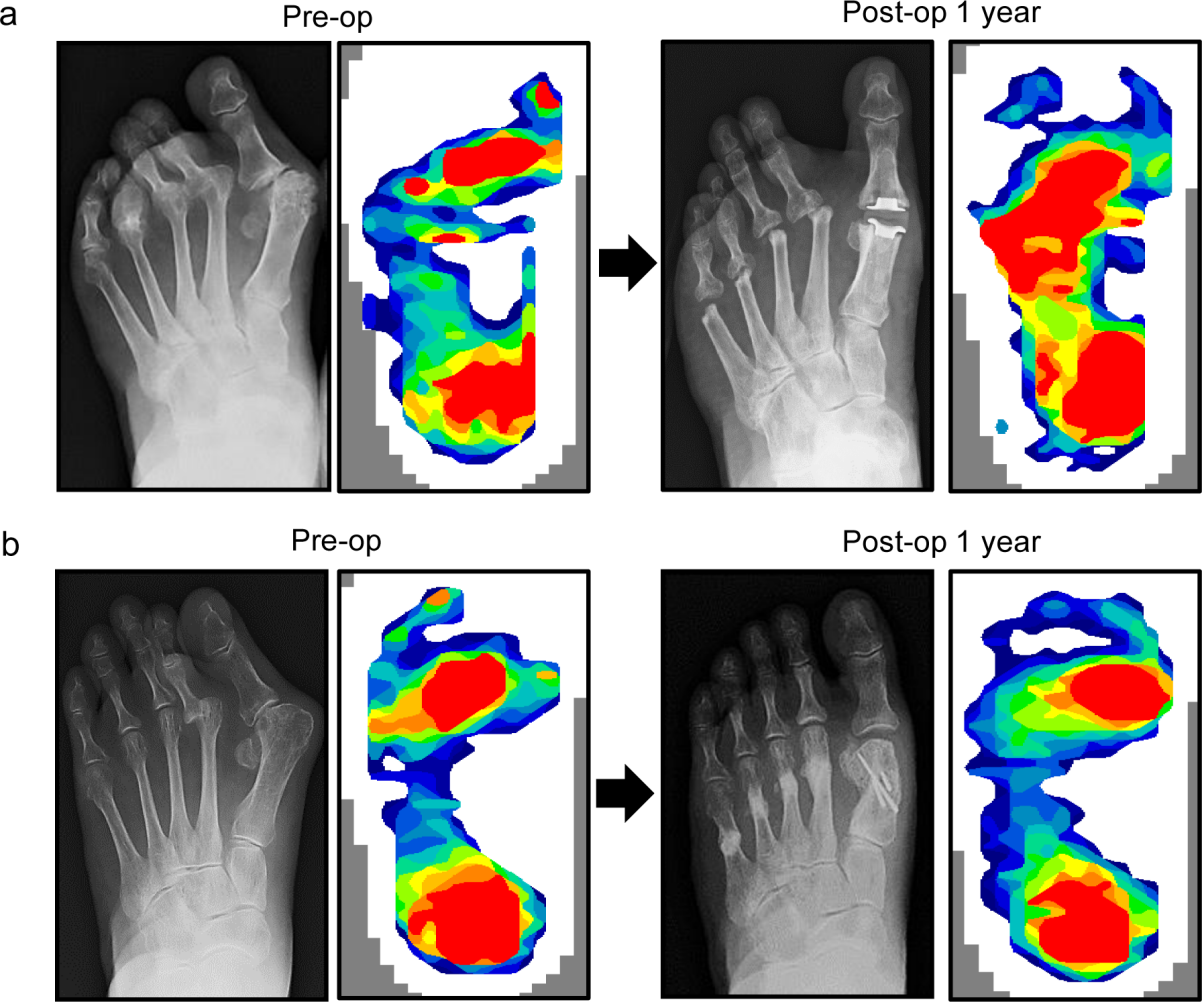
Representative pre-operative and post-operative radiographs and plantar peak pressure distributions of each group. (a) Metatarsal head resection-replacement arthroplasty and (b) metatarsophalangeal joint-preserving arthroplasty.

**Fig 4:**
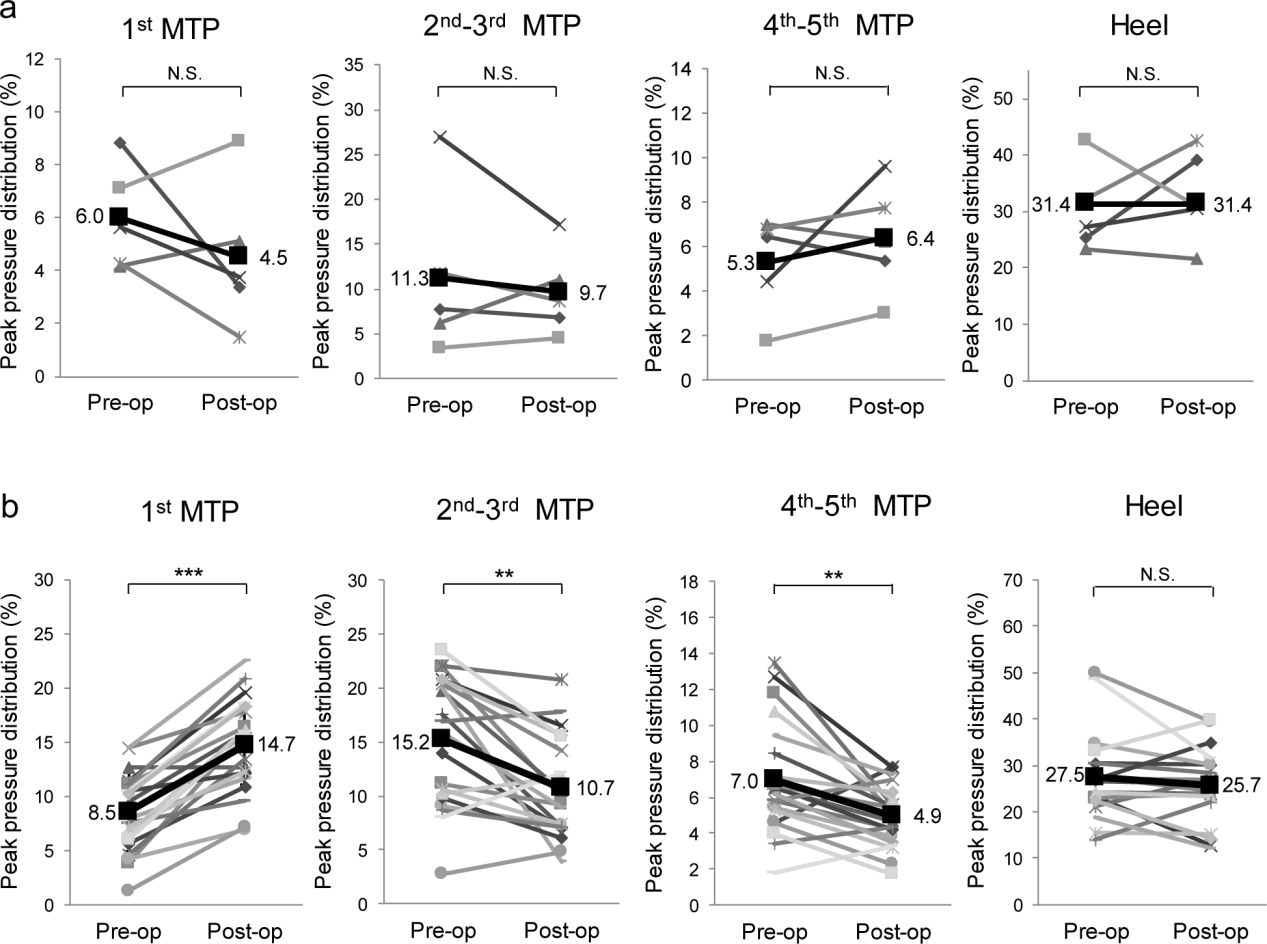
Longitudinal changes in the plantar peak pressure distribution (%) between pre-operation and post-operation of each group. (a) Metatarsal head resection-replacement arthroplasty and (b) metatarsophalangeal joint-preserving arthroplasty. The bold line and number indicate mean values. N.S. (not significant), ** P < 0.01, and *** P < 0.001 pre-operation vs post-operation.

Then, longitudinal analysis of plantar pressure before and after the forefoot operation was performed. Representative X-ray and plantar peak pressure distributions of both surgery groups are shown in Fig 3. In the resection-replacement group, peak pressure distribution was shifted to the lateral side after the operation (Fig 3A). However, in the joint-preserving group, peak pressure distribution was shifted to the medial side, and 1^st^ MTP joint pressure was restored after the operation (Fig 3B). Among the operated RA patients, 5 feet of the resection-replacement group and 20 feet of the joint-preserving group were evaluated longitudinally (Fig 4). Although the number is relatively small, the resection-replacement group showed no significant changes in plantar pressure distribution (Fig 4A). On the other hand, the joint-preserving group showed a significant increase in the 1^st^ MTP joint (8.5% vs. 14.7%; P<0.001) and decrease in 2^nd^-3^rd^ MTP joint (15.2% vs. 10.7%; P<0.01) and 4^th^-5^th^ MTP joint (7.0% vs. 4.9%; P<0.01) pressure distributions after the operation (Fig 4B).

## Discussion

As far as we know, this is the first report to demonstrate the correlations between walking plantar pressure distribution and patient-based outcomes, as well as the differences between the resection-replacement and joint-preserving surgical procedures for RA.

A previous report demonstrated that the first metatarsal head consistently bears the highest load of the other toes in normal feet [26], although Stokes et al. also mentioned that there was considerable variability in the loading distribution of healthy feet [27]. On the other hand, hallux valgus was associated with reduced medial side loading compared with that of healthy feet [27], and hallux valgus patients also demonstrated increased peak pressure under the lateral metatarsal heads that actually increases following resection arthroplasty of the hallux (Keller procedure) [26]. Moreover, another report showed that silastic arthroplasty did not carry high loads when used to treat hallux valgus [27]. Concerning RA, forefoot joint damage was significantly correlated with forefoot pressure [11], and RA patients showed lower medial and higher lateral forefoot peak pressures compared to healthy controls [10]. Taken together, RA forefoot deformity including hallux valgus may be associated with decreased 1^st^ MTP joint loading, and lesser toe deformity may be associated with increased lateral MTP joint loading. Correcting hallux valgus with preservation of the first metatarsal head may be beneficial in restoring 1^st^ MTP joint function and loading, since the first metatarsal head is relatively large, and replacement by a silastic implant may lead to insufficient loading because of the loss of metatarsal head volume.

On the other hand, Woodburn et al. reported that, in hindfoot valgus deformed RA, peak pressure was shifted to the medial forefoot [9], and we have recently reported that hindfoot valgus deformity was associated with higher 1^st^ MTP joint loading and less forefoot pain in RA [19]. In the present study, there were no significant correlations between the leg-heel angle and the 1^st^ MTP joint or 2^nd^-3^rd^ MTP joint peak pressures, suggesting that the influence of forefoot deformity and operation may exceed that of hindfoot deformity in plantar pressure distribution, and 1^st^ MTP joint loading may lead to decreased forefoot pain.

Finally, a longitudinal study showed that joint-preserving arthroplasty may increase 1^st^ MTP joint loading and decrease 2^nd^-3^rd^ MTP joint and 4^th^-5^th^ MTP joint loading, which were associated with the patient-based pain and pain-related score and the shoe-related score. Loss of joint function owing to the dislocation of the proximal phalanges is considered a primary cause of painful plantar callosities of MTP joint [28]. In addition, hammer and claw toe deformities of the lesser toes are often associated with painful dorsal callosities in the PIP joint with low instep shoes [29]. We have previously reported that joint-preservation resulted in a lower HV angle and less MTP joint subluxation or dislocation than resection-replacement, which may be reflected in the better outcomes on SAFE-Q [18].

There are several limitations in the present study. First, patients in the control group were younger and included more males than RA groups, which may be incomparable. Second, although fair clinical outcomes of hallux MTP joint arthrodesis with metatarsal head resection of lesser toes have been reported, this method was not included in this study because of the small number of patients. Third, the selection of methods was dependent on each surgeon’s discretion and not randomized. Fourth, the operation methods in each group were not completely integrated. Fifth, since we are mainly performing joint-preserving arthroplasty recently, the number of patients in the longitudinal study of the resection-replacement group was relatively small.

## Conclusions

The joint-preserving arthroplasty resulted in higher plantar pressure distribution of the 1^st^ MTP joint and lower plantar pressure distribution of the 2^nd^-3^rd^ MTP joint, which were associated with better patient-based outcomes than resection-replacement arthroplasty.

## Supporting information

S1 Table (CSV)
